# Pre-adaptative and adaptative management of multimorphic cancer pain: the keys to optimizing the patient's journey

**DOI:** 10.3389/fpain.2025.1574254

**Published:** 2025-05-20

**Authors:** Julie Fulcrand, Hélène Dewaele, Guillaume Gourcerol, Florian Scotté, Denis Dupoiron, Alexis Burnod, Antoine Lemaire

**Affiliations:** ^1^ Oncology Unit, Valenciennes General Hospital, Valenciennes, France; ^2^ Supportive Care Unit, Valenciennes General Hospital, Valenciennes, France; ^3^ CIC-CRB 1404, Department of Physiology, Univ Rouen Normandie, INSERM, Normandie Univ, ADEN UMR1073, Nutrition, Inflammation and microbiota-gut-brain axis, CHU Rouen, Rouen, France; ^4^ Interdisciplinary Cancer Course Department, Gustave Roussy Cancer Institute, Villejuif, France; ^5^ Department of Anesthesiology, Integrated Center of Oncology, Angers, France; ^6^ Department of Supportive and Palliative Care, Institut Curie, Paris, France; ^7^ Oncology and Medical Specialties Department, Valenciennes General Hospital, Valenciennes, France

**Keywords:** cancer pain, multimorphic pain, supportive care, interdisciplinarity, multimodal management, integrated model

## Abstract

The number of patients living with cancer has increased and their management has dramatically changed, resulting in major survival improvement. Thus a new paradigm arose with a focus not only on cancer treatments but also on maintenance of the best possible quality of life. Cancer pain is frequent and remains insufficiently relieved, highlighting the gap between theory and real life, scientific skills, and their application. Cancer pain is multimorphic, complex, multifaceted, and changes over time from diagnosis until cure or palliative situations. These modifications result from the interaction of intrinsic and extrinsic factors that create disruptions along the cancer care pathway. Pain screening must be systematic, and performed by any healthcare professional in contact with cancer patients at any time, in any context. Pain management must be individualized and adapted to each patient, anticipated whenever possible by identifying disruptive factors. The classical stepwise process should be abandoned in favor of an integrated model where supportive care and, in particular, pain management, is an integral part of cancer care from diagnosis to survivorship. Interdisciplinary management is necessary, requiring efficient teamwork led by a conductor. As supportive care plays a key role, it must be implemented in an early and timely manner, taking into account different aspects of the patient's life including physical, psychological, social, and spiritual aspects.

## Introduction

The incidence of cancer is increasing with 19.3 million new cases worldwide in 2020, and a planned estimation of 30 million in 2040 (1). Due to major therapeutic improvements, life expectancy has dramatically increased, even in advanced and metastatic settings, with cancer becoming a chronic condition in many cases. More than 18 million Americans were cancer survivors in 2022 (2) and their prevalence in Europe ranges from 650 to 1,100/100 000 (3). Thus, a new paradigm arose with a focus not only on cancer treatments but also on the maintenance of the best possible quality of life.

Pain is a common symptom in cancer. Around 39%, 55%, and 66% of patients experience pain after curative treatment, during treatment, and at the advanced stage of the disease respectively (4). Regrettably, these figures are still similar to those published in a prior meta-analysis published 9 years before (5) and a recent update showed that 44.5% of cancer patients overall experience pain (6). Among cancer survivors, analgesics are prescribed to only one-third of those who report pain (7). Finally, cancer-related neuropathic pain, which can affect up to 40% of patients is still underdiagnosed in 33% of cases (8) and sensitization underlying nociplastic pain has to be considered.

Yet, efficient therapies exist, along with detailed recommendations that can help clinicians select and implement the right strategy, for the right patient, at the right time (9–11). However, theoretical knowledge is mandatory but insufficient and even well-designed guidelines implementation strategies fail to improve pain outcomes (12), highlighting the gap between theory and real life, scientific skills, and their application (13).

Thus, one may wonder why so many patients still suffer from cancer pain and what are the barriers to appropriate management. Lack of awareness of patients’ symptoms? Insufficient training and shortage of specialized healthcare professionals? Organizational issues? Compartmentalization of healthcare and dysfunctions in coordination along patient's pathway? Health policy issues?

Pain belongs to the plethora of symptoms associated with cancer and as such, should be managed in the setting of “global care”. There have been numerous debates regarding defining palliative care, supportive care, or survivorship care (14). While the former carries an “end-of-life” connotation and the latter is limited to cured patients, supportive care encompasses the continuum of cancer patient's journey, as it consists of “prevention and management of the adverse effects of cancer and its treatment” (15). Early supportive care has demonstrated significant benefits in cancer patients, but mostly in advanced and palliative cancer situations (16–18). As an integral part of modern oncology, early and timely supportive care is the most adapted setting to deal with pain, whether related to cancer, cancer treatments, comorbidities, or other causes (10, 19). Nevertheless, whatever the word is, especially in a post-COVID era, the priority is to accompany the patient from diagnosis, to prevent or reduce as far as possible the number and severity of painful episodes.

We propose thoughts and ways for improving cancer pain management from a practical perspective.

## Cancer pain has changed: the need for understanding its multimorphism

Along with the increased complexity of cancer management, cancer pain has become more complex and multifaceted, defining the concept of multimorphic cancer pain: “cancer pain is not a fixed entity. It changes, alters, evolves or devolves, and presents in different forms at different periods from diagnosis until cure or palliative situations when applicable. These modifications result from interaction of intrinsic and extrinsic factors that create disruptions, which in turn destabilize pain management” (10, 13).

This concept integrates the heterogeneity (clinical presentation, mechanisms, cause, patient's experience, underlying cancer, environmental factors) and the dynamics of cancer pain integrating intrinsic and extrinsic disruptive factors which can break analgesic balance (cancer evolution, treatments, comorbidities, complications, environmental changes) (10, 13). This high interpatient and intrapatient variability of pain adds tremendous complexity and requires a targeted, personalized, and multimodal approach.

## The need for anticipation

**Figure 1:**
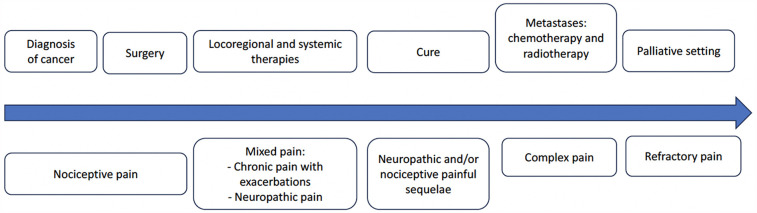
An example of pain evolution in cancer patients.

In cancer patients, painless periods can alternate with phases of acute or chronic pain, paralleling the evolution of the disease, and cancer treatments interspersed with disruptions. Schematically, different factors can be associated: pain due to cancer and its extension—treatment-related pain (which can recur at each treatment period and remain after completion even in cured patients)—pain at progression/relapse—end-of-life pain (Figure 1).

### Predictive factors and predefined pain management plans for targeted patients

Pain prevalence depends on the stage of disease: it can be the lowest in treatment-naïve patients, intermediate during curative therapy, and the highest at advanced stage of disease; pain severity is the lowest after curative treatment and the highest in palliative setting (6). Some cancers are known to be more painful than others, especially at an advanced stage where pancreatic and pelvic cancers often provoke intractable pain. Other risk factors include individual patients’ characteristics, notably emotional and psychological features (underlying anxiety and depression), comorbidities and performance status, type of cancer therapy, and more generally, all events that can exacerbate pain by creating disruptions, whether they are related to cancer itself, cancer treatment, emotional or cognitive aspects (10). All these elements should be detected to anticipate episodes of pain, which can take different forms depending on triggering events (20). Moreover, tools can be used, such as the Cancer Pain Prognostic Scale to predict the probability of pain relief in patients with moderate to severe pain, allowing identification of patients with poor pain prognosis (21).

Thus, it is often possible to foresee the risk of important pain. In this respect, mid/long-term or temporary requirements for challenging pain treatment can be predicted (Table 1). In these targeted patients, anticipation allows early discussions amongst stakeholders to establish an advanced pain management plan, that can be prepared early and implemented as needed.

**Table 1:** Examples of advanced pain management planning.

Situation	Planned strategy
Spinal metastasis with instability	Preventive cementoplasty
Advanced pancreatic adenocarcinoma	Consider the possibility of interventional techniques like intrathecal analgesia
Initiation of radiochemotherapy in a patient with head and neck cancer	Preventive treatment of oral mucositis by photobiomodulation
Initiation of radiotherapy in a woman with breast cancer and risk factors for dermatitis	Preventive treatment of radiodermitis by photobiomodulation
Initiation of neurotoxic chemotherapies	Preventive treatment of chemotherapy-induced peripheral neuropathy by photobiomodulation, early implementation of antineuropathic treatments and rehabilitation Early identification of chemobrain and cognitive rehabilitation
Painful cancer surgery	Around the clock and breakthrough pain medications

In contrast, unpredictable pain episodes can occur (complication, intercurrent event) that require adaptability and rapid execution of a new strategy. Emergencies with breakthrough uncontrolled acute pain must be managed appropriately, if necessary in dedicated emergency pain departments: search for a causal etiology (related to the cancer, its treatments, or not) that could be treated medically or surgically, quick relief of pain using appropriate medications or techniques, identification of potential disruptive elements and reevaluation of the patient's healthcare path (20).

In any case, timely intervention requires adequate pain screening, which should be systematic at each step of the patient's journey.

## The need for continuous evaluation of patients

### When?

Patients do not always report pain spontaneously, either because they consider that suffering is normal in cancer, or they do not dare complain, or they do not expect any possible relief. Therefore, the question of pain must systematically be raised, in an environment of benevolent and attentive listening. In case of a positive answer, pain must be further characterized, in particular, to identify a possible neuropathic component, and severity must be measured using an easy-to-use and reproducible tool such as visual analogic scale (VAS) or numerical rating scale (NRS) (9, 22) (Table 2). The possibility of analgesics de-escalation of analgesics must be considered.

**Table 2:** Pain screening and management.

Pain screening	
Who?	All cancer patients
When?	Throughout the whole journey from diagnosis to survivorship
By whom?	Any healthcare professional
In which setting?	Ambulatory or hospital visits, oncology-driven or not
How?	- Usual easy-to-use scales (VAS, NRS) for basic screening - Multidimensional questionnaires for comprehensive characterization of pain - ePROMs for early alerts - Prescription Opioid Misuse Index scale (POMI)
Pain management	
Easily manageable pain	Oncologist, general practitioner, advanced practice nurse, pharmacist, patient/caregiver
Breakthrough episodes of acute uncontrolled pain	Emergency pain referral structure
Chronic severe/refractory pain	Pain specialists and multidisciplinary teams who can propose alternative/interventional techniques
Complex situations	Multidisciplinary teams

### By whom?

Any healthcare professional, in the hospital or ambulatory setting, even in non-oncologic consultations, must ask about pain at any contact with the patient, to raise alerts that will allow early management: general practitioner, oncologist, surgeon, radiotherapist, pharmacist, nurse, among others.

In addition, as soon as the diagnosis of cancer is notified, patient must be informed pain should never be concealed because it might have deleterious effects and can jeopardize appropriate cancer management, while efficient analgesic therapies can be administered. The patient (and/or caregiver) should be educated to be a whistle-blower who triggers emergency management of acute pain, a source of information for the physician to orient treatment of chronic pain, and also an autonomous actor of everyday pain management. Information must also be provided to patients about misuse and overdosing that can provoke side effects.

### How?

Pain being a subjective symptom, only patient-reported outcome measures (PROMs) are relevant for evaluation, except for patients unable to report their feelings such as neonates and infants, patients with altered consciousness or severe psychiatric diseases for whom other means of assessment exist (23, 24). However, since pain is multidimensional, in-depth evaluation is complex and, in addition to basic tools, can also include multidimensional scales such as the Brief Pain Inventory or the McGill Pain questionnaire (23). Consequences of pain and quality of life must be part of the overall assessment. In our digital world, e-PROMs can be beneficially used for longitudinal follow-up of pain, to further optimize its management thanks to timely reporting of symptoms allowing early interventions. The benefits of PROMs and e-PROMs in cancer patients have been widely demonstrated in randomized clinical trials and real-life studies (24).

## The need for timely and adapted pain management

### Therapeutic options

Pain management should cover every aspect of cancer pain using an analytic approach, starting with the identification of all determinants including non-clinical ones (e.g., understanding why an analgesic balance is broken (13). The cause of pain should be investigated without preconceived ideas (pain in a cancer patient is not always cancer-related but must be considered as a possible cancer symptom) to carry out etiological treatment as far as possible (9). Due to its multimorphic nature, cancer pain requires customized, systemic, and multimodal management, including pharmacological, and non-pharmacological therapies (e.g., acupuncture, hypnotherapy, music therapy, psychotherapy) (9, 25, 26). If patients become refractory to conventional treatments, targeted interventional therapies (e.g., photobiomodulation, external beam radiotherapy, surgery, intrathecal drug delivery, neuromodulation, or nerve blocks) can be used to provide relief (9, 27).

### Strategy

This global management can only be achieved by an integrative, holistic yet specialized approach (13).

**Figure 2:**
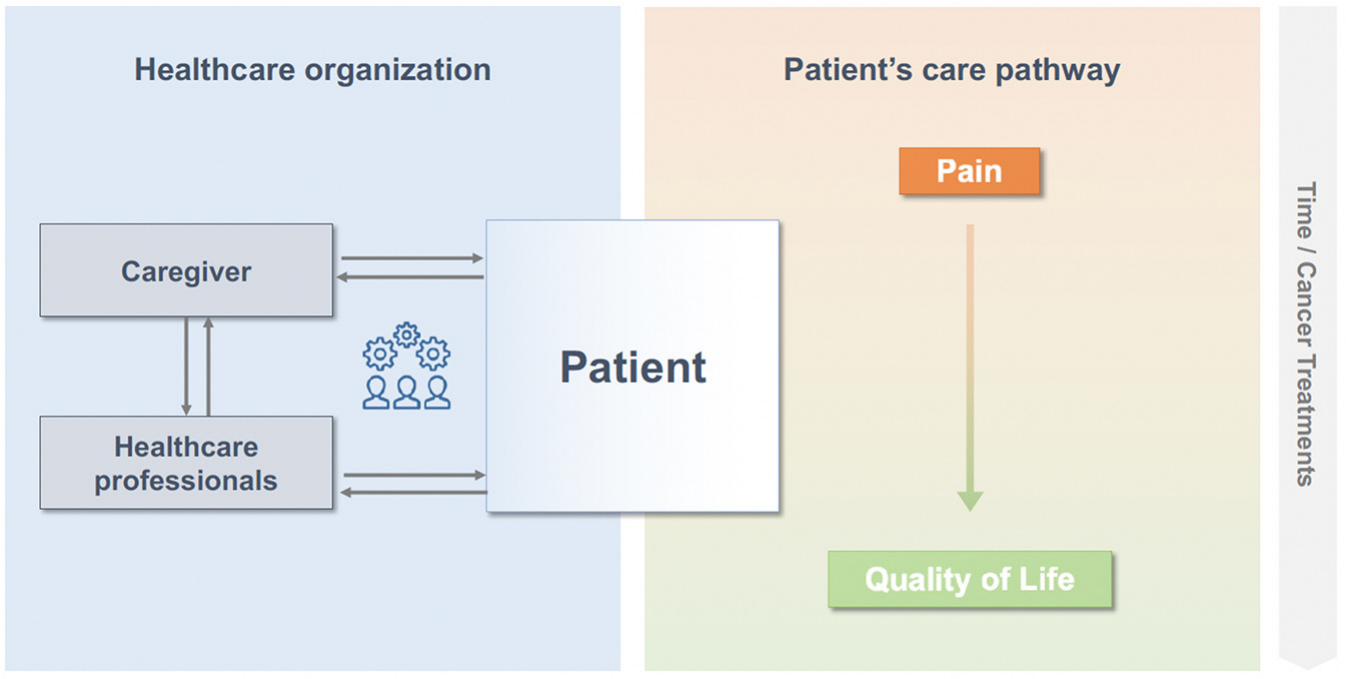
An integrated approach.

From a strategic perspective, the classical stepwise process should be abandoned in favor of an integrated model where supportive care, and in particular, pain management, is an integral part of cancer care from diagnosis to survivorship (Figure 2). Cancer pain should no longer be managed as an entity, but rather a multidimensional suffering component properly assessed and managed by an interdisciplinary care team (28).

Several models have been developed to conceptualize and improve palliative care, some of which apply to pain management, such as integrated models, with active collaboration between oncologist and supportive care team, providing comprehensive and personalized care (29, 30). Key elements include a team-based approach and the use of targeted and timely treatments (18).

## The need for teamwork

Multidisciplinarity consists of an additional, not integrative, juxtaposition of disciplines; interdisciplinarity analyzes, synthesizes, and harmonizes links between disciplines into a coordinated and coherent aggregate; transdisciplinarity integrates natural, social, and health sciences in a humanities context, and transcends their traditional boundaries, providing holistic schemes and looking at dynamics of the whole system (31). The objectives of these approaches are resolve complex problems, to provide different perspectives, and to deliver comprehensive health services.

Since they can be cumbersome to manage, they should be used appropriately, with the right people, at the right time, for the right purpose. Based on the Canadian model (32), the need for multi/interdisciplinarity increases with the increasing complexity of the situation. While it is mandatory in some key periods of patients’ management (diagnosis, relapse, complex situations), it is useless in eventless phases. Thus, key periods requiring multi/interdisciplinarity should be identified, which reflects the need for flexibility in cancer pain management, and highlights the key role of a leader or conductor who evaluates the needs and coordinates the whole team. Depending on the country's healthcare organization, it can classically be oncologist, general practitioner, nurse, or another healthcare professional. However, supportive care physician seems to be most relevant to take this role. Dedicated regional multidisciplinary meetings can be organized on a weekly or monthly basis to review complex cases and make decisions on treatment.

Multi/interdisciplinarity implies teamwork, which in turn requires that people are willing to work together cooperatively and effectively (33). But physicians as well as most healthcare professionals are not trained for teamwork and this should be part of initial and continuous education. The established reputation of medical individualism is contrary to the concept of collectivity which underpins teamwork (33), but to the credit of physicians, cancer management being increasingly sophisticated and complex requires increasing specialization and partitioning of tasks. Another condition for the effectiveness of teamwork is that the role, responsibilities, and intervention area of each team member be clearly defined.

Finally, one of the pillars of teamwork is communication, which is also a field where there is considerable scope for progress to be made.

## Practical issues

Optimal cancer pain management requires skilled human resources (including but not limited to pain specialists, nurses, pharmacists, psychologists, and physiotherapists) and appropriate facilities. For instance, interventional techniques can be delivered only in highly specialized sites with expert healthcare teams, technical support, and specific organization (27). These expert sites should be fairly distributed to avoid important territorial inequalities and difficulties in healthcare access for some populations. The same applies to pain emergency departments and specialized pain management units. Unfortunately, administrative constraints can jeopardize smooth functioning since within hospitals or geographical areas, budgets are fragmented and funding dedicated to a given activity cannot be transferred to another department. Overall costs can be probably reduced by the implementation of early supportive care that avoids disruptions in the cancer care pathway. Telemedicine can solve some but not all issues, by allowing first-line management in areas devoid of pain units.

## Conclusion

Thanks to the vast panel of therapeutic options available, multimorphic cancer pain can be relieved in most patients. However, to achieve this objective, the way of thinking must change. Cancer pain is a fluctuating and multimorphic entity, influenced by intrinsic and extrinsic factors that must all be understood, taken into account, and addressed. Pain must be a constant concern of any healthcare professionals who deal with cancer patients, and it should be managed using an integrative and dynamic approach, adapted to variability of patients’ needs. Communication and teamwork should be developed in healthcare professionals as they are major skills for multidisciplinary management (Table 3).

**Table 3:** Highlights.

• Cancer pain has changed, it has become multimorphic and more complex • Supportive care is crucial at each step of cancer patients’ journey • Timely and tailored cancer pain management requires anticipation, exhaustive as well as continuous evaluation, multimodal approach, and interdisciplinarity

## Data Availability

The original contributions presented in the study are included in the article/Supplementary Material, further inquiries can be directed to the corresponding author.
